# Supplementary material for: P-TRAP: a Panicle Trait Phenotyping tool
Source: BMC Plant Biol. 2013 Aug 29;13:122. doi: 10.1186/1471-2229-13-122 (PMC3848748; doi:10.1186/1471-2229-13-122)
Supplement: Additional file 7 — P-TRAP source code. The Netbeans project that contains the source code of the application. [file 1471-2229-13-122-S7.zip › javadoc.zip!/javadoc/org-netbeans-modules-riceoptions/index-all.html]

Index (Rice Options)


JavaScript is disabled on your browser.


- Package
- Class
- Tree
- Deprecated
- Index
- Help

*org.netbeans.modules.riceoptions 1.0*

- Prev
- Next

- Frames
- No Frames

- All Classes

A C G I O R U 


## A

addPropertyChangeListener(PropertyChangeListener) - Method in class org.netbeans.modules.riceoptions.RiceOptionsOptionsPanelController


applyChanges() - Method in class org.netbeans.modules.riceoptions.RiceOptionsOptionsPanelController


## C

cancel() - Method in class org.netbeans.modules.riceoptions.RiceOptionsOptionsPanelController


## G

getComponent(Lookup) - Method in class org.netbeans.modules.riceoptions.RiceOptionsOptionsPanelController


getHelpCtx() - Method in class org.netbeans.modules.riceoptions.RiceOptionsOptionsPanelController


## I

isChanged() - Method in class org.netbeans.modules.riceoptions.RiceOptionsOptionsPanelController


isValid() - Method in class org.netbeans.modules.riceoptions.RiceOptionsOptionsPanelController


## O

org.netbeans.modules.riceoptions - package org.netbeans.modules.riceoptions


## R

removePropertyChangeListener(PropertyChangeListener) - Method in class org.netbeans.modules.riceoptions.RiceOptionsOptionsPanelController


RiceOptionsOptionsPanelController - Class in org.netbeans.modules.riceoptions
:   The handler of the rice options dialog.

RiceOptionsOptionsPanelController() - Constructor for class org.netbeans.modules.riceoptions.RiceOptionsOptionsPanelController


## U

update() - Method in class org.netbeans.modules.riceoptions.RiceOptionsOptionsPanelController

A C G I O R U

- Package
- Class
- Tree
- Deprecated
- Index
- Help

*org.netbeans.modules.riceoptions 1.0*

- Prev
- Next

- Frames
- No Frames

- All Classes
